# Supplementary material for: A fast-acting inhibitor of blood-stage P. falciparum with mechanism distinct from artemisinin and chloroquine
Source: bioRxiv. 2024 Aug 12:2024.08.12.607553. Preprint. [Version 1] doi: 10.1101/2024.08.12.607553 (PMC11343144; doi:10.1101/2024.08.12.607553)
Supplement: Supplement 1 [file NIHPP2024.08.12.607553v1-supplement-1.pdf]

bioRxiv preprint doi: https://doi.org/10.1101/2024.08.12.607553; this version posted August 12, 2024. The copyright holder for this preprint (which was not certified by peer review) is the author/funder, who has granted bioRxiv a license to display the preprint in perpetuity. It is made available under aCC-BY 4.0 International license.

Supporting Information

Figure S1. MMV1580853 inhibition of parasite growth
A. Structure of MMV1580853
B-C. 72h dose-dependent growth inhibition of blood-stage *P.falciparum* strains: (B) chloroquine-resistant W2 and chloroquine-susceptible 3D7; (C) parent Dd2 (background strain for Kelch13 mutants) and parent NF54^{pCRISPR} (background strain for TetDOZI KD strains). Results are the mean and 95% CI of three independent experiments after subtraction of background fluorescence of uninfected RBCs. Growth is normalized relative to untreated controls.

bioRxiv preprint doi: https://doi.org/10.1101/2024.08.12.607553; this version posted August 12, 2024. The copyright holder for this preprint (which was not certified by peer review) is the author/funder, who has granted bioRxiv a license to display the preprint in perpetuity. It is made available under aCC-BY 4.0 International license.

A

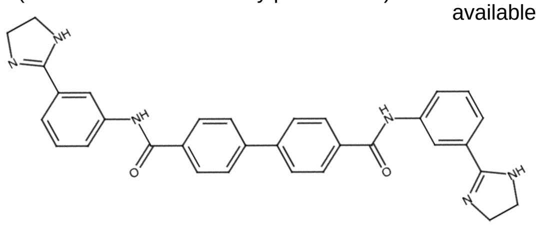

B

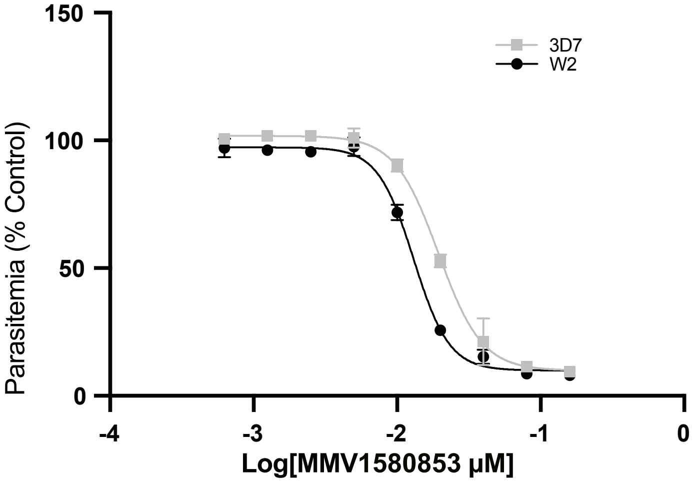

C

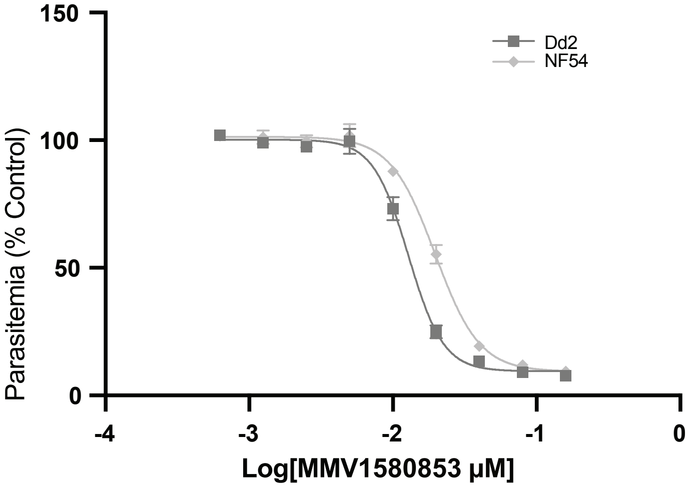

Figure S1. MMV1580853 inhibition of parasite growth

A. Structure of MMV1580853

B-C. 72h dose-dependent growth inhibition of blood-stage *P.falciparum* strains: (B) chloroquine-resistant W2 and chloroquine-susceptible 3D7; (C) parent Dd2 (background strain for Kelch13 mutants) and parent NF54^{pCRISPR} (background strain for TetDOZI KD strains). Results are the mean and 95% CI of three independent experiments after subtraction of background fluorescence of uninfected RBCs. Growth is normalized relative to untreated controls.
